# Design and implementation of decision support for tobacco dependence treatment in an inpatient electronic medical record

**DOI:** 10.1186/1748-5908-10-S1-A1

**Published:** 2015-08-14

**Authors:** Steven L Bernstein, June Rosner, Michelle DeWitt, Allen Hsiao, James Dziura, Benjamin Toll

**Affiliations:** Department of Emergency Medicine, Yale School of Medicine, Yale University, New Haven, CT 06519 USA; Yale School of Medicine, Yale-New Haven Hospital, New Haven, CT 06510 USA

## Background

Tobacco dependence treatment for hospitalized smokers results in long-term cessation if treatment continues at least 30 days post-discharge. Methods to leverage inpatient interventions into post-hospitalization care are unclear; health information technology may facilitate ongoing treatment.

## Objective

To develop and test an order set and best practice alert (BPA) addressing tobacco dependence treatment for hospitalized smokers embedded in an electronic medical record (EMR).

## Methods

A 2-arm randomized clinical trial of 250 physicians and patients treated by those physicians. The physician is the unit of randomization. A BPA and order set were developed and embedded in the Epic (Madison, WI) EMR used at 2 hospitals. When an adult patient is admitted to a medical service, a BPA fires if the patient is coded in the EMR as a smoker. For physicians randomized to the intervention, the BPA offers to take the physician to an order set to prescribe tobacco treatment medications and refer the patient to the state smoker's quitline. Additionally, tobacco use disorder" is added to the patient's problem list, and an email is sent to the patient's primary care provider (PCP). In the control arm, a BPA silently fires with no additional actions for the physician.

## Results

Since August 2013, the BPA fired for 4187 patients (1887 intervention, 2300 control). Compared to control arm physicians, intervention physicians were more likely to order tobacco treatment medication (24% v. 9%, P = 0.0001, Fisher's exact test), populate the problem list with tobacco use disorder (46% v. 4%, P < 0.0001), and make a referral to the state smokers' quitline (19% v. 0%, P < 0.0001). In addition, intervention physicians sent an email to the patient's PCP 1876 (99%) times.

## Conclusion

Designing and implementing an order set and BPA for tobacco treatment in an EMR is feasible and acceptable. Data on cessation outcomes are pending.

